# Marker-Assisted Introgression of *Saltol* QTL Enhances Seedling Stage Salt Tolerance in the Rice Variety “Pusa Basmati 1”

**DOI:** 10.1155/2018/8319879

**Published:** 2018-02-11

**Authors:** Vivek Kumar Singh, Brahma Deo Singh, Amit Kumar, Sadhna Maurya, Subbaiyan Gopala Krishnan, Kunnummal Kurungara Vinod, Madan Pal Singh, Ranjith Kumar Ellur, Prolay Kumar Bhowmick, Ashok Kumar Singh

**Affiliations:** ^1^ICAR-Indian Agricultural Research Institute, Division of Genetics, New Delhi 110012, India; ^2^Banaras Hindu University, School of Biotechnology, Varanasi 221005, Uttar Pradesh, India; ^3^ICAR-Indian Agricultural Research Institute, Division of Plant Physiology, New Delhi 110012, India; ^4^Rice Breeding and Genetics Research Centre, ICAR-Indian Agricultural Research Institute, Aduthurai 612 101, India

## Abstract

Marker-assisted selection is an unequivocal translational research tool for crop improvement in the genomics era. Pusa Basmati 1 (PB1) is an elite Indian Basmati rice cultivar sensitive to salinity. Here, we report enhanced seedling stage salt tolerance in improved PB1 genotypes developed through marker-assisted transfer of a major QTL, *Saltol*. A highly salt tolerant line, FL478, was used as the *Saltol* donor. Parental polymorphism survey using 456 microsatellite (SSR)/QTL-linked markers revealed 14.3% polymorphism between PB1 and FL478. Foreground selection was carried out using three *Saltol*-linked polymorphic SSR markers RM8094, RM493, and RM10793 and background selection by 62 genome-wide polymorphic SSR markers. In every backcross generation, foreground selection was restricted to the triple heterozygotes of foreground markers, which was followed by phenotypic and background selections. Twenty-four near isogenic lines (NILs), with recurrent parent genome recovery of 96.0–98.4%, were selected after two backcrosses followed by three selfing generations. NILs exhibited agronomic traits similar to those of PB1 and additional improvement in the seedling stage salt tolerance. They are being tested for per se performance under salt-affected locations for release as commercial varieties. These NILs appear promising for enhancing rice production in salinity-affected pockets of Basmati Geographical Indication (GI) areas of India.

## 1. Introduction

Rice plants suffer severe salt injury in both seedling and reproductive stages; the most common damages are attributed to osmotic imbalance, membrane destabilisation, and failure of photosynthetic machinery [1]. The damage due to salt stress is often cumulative as the seedling stage sensitivity leads to poor crop establishment, and reproductive stage sensitivity results in reduced yields [2]; the combined effect of damages at both the stages may lead to total crop loss. Nevertheless, seedling stage tolerance can sustain crop production in salinity prone areas by promoting good initial establishment leading to healthy vegetative growth that can augment crop yield [3]. There are some saline ecosystem-adapted traditional rice landraces such as Pokkali and Nona Bokra that are known to be salt tolerant. Salt tolerance in rice is manifested through morphological, physiological, and metabolic responses that includes stomatal changes, sodium exclusion, tissue tolerance, apoplastic salt compartmentalization, salt sequestration into older tissues, and regulation of the antioxidants [2–5]. Apart from the understanding of physiological and metabolic responses to salt stress, quantitative trait loci (QTLs) and genes governing salt tolerance have also been reported in rice. These include a major QTL, *Saltol* identified on chromosome 1 of Pokkali, and *SKC1* (*OsHKT1;5*), a gene located within the *Saltol* region identified from Nona Bokra. The QTL *Saltol* imparts salt tolerance by regulating Na^+^/K^+^ homeostasis under salt stress [6–9].

In India, of the estimated area of 7.0 million ha (mha) occupied by saline soils, a sizeable fraction occurs in the Indo-Gangetic plains covering the states of Haryana, Punjab, Uttar Pradesh, Rajasthan, and Bihar [10]. Basmati rice is exclusively grown in an area of over 1.68 mha spanning the Indo-Gangetic plains; this region is recognised as its Geographical Indication (GI) area [11–14]. In recent times, soil salinity has become a major problem affecting Basmati rice cultivation, especially in the state of Haryana [11]. Haryana has about 1.0 mha under Basmati rice, majority of which is threatened by inland salinity resulting from the continuous use of brackish irrigation water [12]. None of the popular Basmati cultivars is reported to be tolerant to salt stress.

Basmati rice is preferred globally for its aromatic grains with unparalleled cooking qualities [11] such as extra-long slender grains, rich aroma, white kernels, translucent endosperm, high cooking elongation, fluffy cooked kernels, good palatability, and medium amylose content. Commercially released in 1989 by ICAR-Indian Agricultural Research Institute (ICAR-IARI), Pusa Basmati 1 (PB1) is the first semidwarf and high-yielding Basmati variety in the world. The release of PB1 revolutionized Basmati rice production in India, because of several advantages over the traditional Basmati cultivars: (a) It had an average yield of more than 4.5 t/ha, as against the low average yield of 2.5 t/ha for the traditional cultivars; (b) PB1 was shorter with robust plant stature, and (c) PB1 matured faster than the late and photosensitive traditional Basmati cultivars [15, 16]. Soon after the release, PB1 got established as a premium cultivar and was extensively cultivated. Twenty-eight years after its commercial release, even today, PB1 is cultivated in about 0.16 mha (~10% of the total Basmati area) in India. It is used extensively in Basmati rice improvement programmes as donor for quality traits as well as high yield. However, PB1 is sensitive to several biotic stresses such as diseases (bacterial blight, blast, sheath blight, and bakanae) and pests (brown plant hopper) and also to abiotic stresses, such as soil salinity and drought. PB1 has been improved for resistance to bacterial blight [17], blast [18], and sheath blight [19] using molecular markers as indirect selection tools, but improvement of salinity tolerance of PB1 is yet to be achieved.

In recent times, marker-aided selection (MAS) has been widely acclaimed as the most effective method of transferring desirable traits [1, 8, 20–22] in rice, including salinity tolerance. The conventional breeding efforts for salinity tolerance in rice had limited success, possibly due to their long turnover time, cumbersome screening procedures, and complex genetic control of the trait [1]. For transferring seedling stage salt tolerance, *Saltol* QTL is the only best-known target locus that is amenable to MAS. As the donor for *Saltol*, FL478 (IR 66946-3R-178-1-1), a highly salt tolerant RIL derived from the cross IR29/Pokkali, has been successfully deployed in breeding programmes in many countries. The SSR markers RM3412, AP3206, and RM8094 are used for transfer of *Saltol* [11, 23–26]. Since grain quality traits are of paramount importance, MAS in Basmati rice needs special attention, especially when the transferred gene(s)/QTL(s) are sourced from non-Basmati donors [27, 28]. The *Saltol* donor, FL478, is a non-Basmati line that has grain characteristics such as medium bold shape, red pericarp, chalky endosperm, no aroma, high amylose content, and low gel consistency. The recovery of grain quality is achieved by integrating phenotypic selection for these traits, in every MAS stage [11].

In this paper, we report marker-aided introgression of the QTL *Saltol* from FL478 into PB1 and the resulting improvement in seedling stage salt tolerance of the PB1 near isogenic lines (NILs). Other agronomic features and grain quality of the NILs were comparable to those of the recurrent parent, PB1.

## 2. Materials and Methods

### 2.1. Plant Materials

The parents used in the present study were (a) PB1, as the recurrent parent (RP), and (b) FL478, as the donor parent for *Saltol*. FL478 is a breeding line with very high level of seedling stage salt tolerance; it can endure salt solutions with electrical conductivity (EC) of up to 15 dSm^−1^ for more than a fortnight. Both the parents were first evaluated for tolerance to 100 mM NaCl solution (EC of 11.6 dSm^−1^) at seedling stage to validate their salt tolerance levels before initiating the crossing programme. Salt tolerance was scored using the standard evaluation system (SES) for rice developed by the International Rice Research Institute, Manila, Philippines [29, 30]. In the pre-screening, the recurrent parent, PB1, was found highly sensitive to salt stress and recorded a score of nine, while the donor parent, FL478, was tolerant and recorded a score of one (Supplementary Figure 1). Crosses were made at IARI-Rice Breeding and Genetics Research Centre, Aduthurai, Tamil Nadu (IARI-RBGRC), and subsequent generations were shuttled between the ICAR-IARI, New Delhi, during *Kharif* season and IARI-RBGRC during off-season.

### 2.2. Breeding Strategy

**Figure 1:**
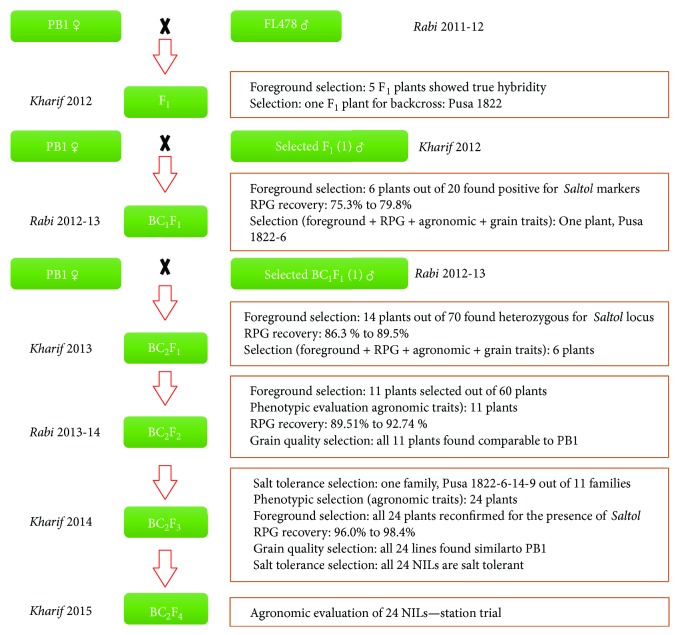
Breeding scheme used in the marker-assisted backcross programme for the transfer of *Saltol* locus in the background of the elite rice variety, Pusa Basmati 1.

PB1 was crossed as the female parent with FL478, and the hybridity of the *F_1_* plants was confirmed using the SSR marker, RM493. The confirmed *F_1_*s were backcrossed to PB1 (always used as the female parent in backcrosses) to generate the *BC_1_F_1_* seeds. The plant selected in *F_1_* was designated as Pusa 1822; the lines derived from the backcross programme (Figure 1) carried the designation as the prefix, for example, Pusa 1822-6-14-9. The parental lines were screened for polymorphism at the target QTL locus using twenty-one *Saltol*-linked SSR markers, of which three markers RM8094, RM493, and RM10793 were found to be polymorphic (Supplementary Figure 2); all the three markers were used for foreground selection. Further, the genome-wide polymorphism between the parents was tested using 435 SSR markers, which identified 62 polymorphic markers that were employed for background selection (Supplementary Table 1). In the *BC_1_F_1_* generation, the plants that tested positive for the three markers used for foreground selection were screened with the 62 SSR markers and subjected to phenotypic evaluation for agronomic traits, including grain characteristics. The plant with the highest recurrent parent genome (RPG) recovery, and having most similarity to the RP, was backcrossed to PB1 to produce the *BC_2_F_1_* seeds. The *BC_2_F_1_* plants were handled in the same manner, except for using only those markers for background selection that were heterozygous in *BC_1_F_1_*. The selected *BC_2_F_1_* plants were selfed to produce *BC_2_F_2_* generation. Each *BC_2_F_2_* plant was subjected to foreground selection to identify plants homozygous for all the three foreground markers. The selected plants were subjected to background and phenotypic selections, the former to assess the recovery of RPG, using markers that were heterozygous in *BC_2_F_1_*, and the later to determine the recovery of Basmati quality traits. The selected *BC_2_F_2_* plants were selfed to raise *BC_2_F_3_* families, which were screened for seedling stage salt tolerance. The family showing the highest level of salt tolerance was transplanted in the field and evaluated for agronomic performance and grain quality. Agronomically superior members of the tolerant family were subjected to foreground screening to confirm the presence of *Saltol* alleles in homozygous state and background selection based on markers that were heterozygous in *BC_2_F_2_* family to assess further increase in the RPG recovery. The salt tolerant lines were advanced to *BC_2_F_4_* generation.

### 2.3. Molecular Analyses

Genomic DNA was isolated from young leaves of the test lines when they were about 40 days old using the standard Cetyl Trimethyl Ammonium Bromide protocol [31]. Polymerase chain reaction- (PCR-) based amplification of the target genomic fragments by the primer pairs for each selected marker was performed in a 10 *μ*l reaction mix constituted by adding 25–30 ng genomic DNA, 5 pmol each of the two primers, 0.05 mM each of the four dNTPs, and PCR buffer (10x) containing 10 mM Tris (pH 8.4), 50 mM KCl, and 1.8 mM MgCl_2_. To this mix, 0.5 U of Taq DNA polymerase was added, and the volume made up to 10 *μ*l using nuclease free water. The PCR was run for 35 cycles comprising of denaturation for one minute at 94°C, followed by annealing for one minute at 55°C, and primer elongation for two minutes at 72°C, sandwiched between an initial denaturation for five minutes at 94°C and the final extension for seven minutes at 72°C. The amplified products were electrophoresed in 3.5% agarose gel, and the products were visualized using a gel documentation system. The marker segregation data was graphically compiled in each generation using Graphical GenoTypes (GGT) version 2.0 software [32].

### 2.4. Marker-Aided Selection

Details of three *Saltol*-linked SSR markers, RM8094, RM493, and RM10793, used for foreground selection such as their physical position on chromosome 1, primer nucleotide sequences, and physical locations within the *Saltol* QTL are given in Supplementary Table 2. The details of all the genome-wide polymorphic SSR markers used for assessing the background polymorphism were sourced from the rice marker database at Gramene (http://www.gramene.org). In each backcross generation, background selection was done after foreground and phenotypic selections. In the background selection process using 62 polymorphic markers, the number of plants with homozygous alleles similar to PB1 and heterozygotes was counted separately for each marker. A reductionist strategy was followed for background selection; markers that became homozygous for the PB1 allele in a given generation were not included in the assay for the subsequent generations. RPG recovery was computed using following formula:

(1) $$\begin{array}{c} RPG\;recovery\%=\left[\frac{number\text{ of }marker\;homozygous\;for\;RP\;alleles\;+\left(0.5\times number\text{ of }heterozygotes\;markers\right)}{total\;number\text{ of }polymorphic\;markers}\right]\times 100. \end{array}$$

To ensure maximum recovery of the carrier chromosome of the *Saltol* QTL, the chromosome 1 was surveyed with 42 evenly distributed SSR markers together with 21 markers linked to the *Saltol* region. Complete recovery of the chromosome 1 together with *Saltol* was specifically targeted, while exercising selections for the background genome.

### 2.5. Screening for Seedling Stage Salt Tolerance

The PB1 NILs homozygous for the *Saltol* QTL along with the two parents were screened for seedling stage salt tolerance at the National Phytotron Facility, ICAR-IARI, New Delhi. Average day/night temperature of approximately 32/25°C and relative humidity of 70–80% were maintained at the screen house throughout the study period. Polystyrene floats with a 14 × 8 matrix of holes lined with a nylon net at the bottom side, and suspended in plastic crates filled with 10 litres of Yoshida nutrient solution [1, 33], were used for screening. The experiment was set up according to a randomized complete block design with two treatments (0.0 mM as control and 100 mM (EC of 11.6 dSm^−1^) NaCl for salt stress) and three replications. Each replication comprised two plastic crates, one crate having six plants each of 12 NILs and the parents PB1 and FL478. The parents served as susceptible and salt tolerant checks. Four-day-old pregerminated seeds were surface sterilized using 70% ethanol and 5% sodium hypochlorite for five minutes each, transferred into the holes in the polystyrene floats, and allowed to germinate over the nutrient solution. The seedlings were subjected to salinity stress after 14 days, starting with an EC of 3 dSm^−1^ by adding 26 mM NaCl concentration in the nutrient solution, and subsequently elevating to 11.6 dSm^−1^ (100 mM NaCl) three days after. The same volume of deionized water was added in the control set. The nutrient solution was replaced once a week, and its pH was maintained daily at 5.8 (adjusted by adding either 1 N NaOH or HCl). The EC of the nutrient solution was recorded daily. Sixteen days after imposing the full salt stress, the symptoms were scored as per SES for rice [30]. The genotypes showing score of 1–3 were classified as tolerant, those with a score of 5 were moderately tolerant, and those with scores of 7–9 were rated as susceptible.

### 2.6. Agronomic and Grain Quality Assessment

Agronomic evaluation of the *BC_2_F_4_* NILs along with both the parents was carried out during *Kharif* 2015 at the research farm of the Division of Genetics, ICAR-IARI, New Delhi, in a field trial laid out in a randomized complete block design with two replications and plot size of 5m^2^. Twenty-five-day-old seedlings were transplanted at a spacing of 20 cm × 15 cm, and the trial was maintained adopting recommended agronomic practices. From each replication, data on various agronomic traits, namely, days to 50% flowering (DFF), plant height (PH), effective tillers per plant (ETP), panicle length (PL), spikelet fertility (SF), weight of 1000 grains (TW), and grain yield per plant (YLD), were recorded from five random plants selected from each entry. The harvested grains from the NILs and their parents were assessed for quality traits like hulling recovery (HUL), milling recovery (MIL), and cooking-related characters, such as kernel length before and after cooking (KLBC and KLAC, resp.), kernel elongation on cooking (ER), alkali spreading value (ASV), and aroma (AROM) as described earlier [34].

### 2.7. Na^+^ and K^+^ Contents in Shoots and Roots

Since *Saltol* acts by balancing the Na^+^ and K^+^ ions in the plant system to counter the salt stress, we estimated the cationic concentrations in shoots and roots from the salt-stressed and salt-unstressed plants of the NILs and the parents [35]. The plant samples were prepared by carefully cleaning the shoots and roots and then drying them at 80°C for 24 h. The dried samples were ground to fine powder in a rotary mill. 500 mg of the powder was then digested in 10 ml of diacid digestion mixture (HNO_3_ and HClO_4_, 9 : 4). The digest was cooled and washed into a volumetric flask, and the volume made up to 50 ml. The mixture was filtered with Whatman number 42 filter paper and analysed for Na^+^ and K^+^ using Systronics Type 128 flame photometer (Systronics India).

### 2.8. Statistical Analyses

The data were analysed for standard statistical tests using the software package Statistical Tools for Agricultural Research STAR 2.0.1 [36].

## 3. Results

### 3.1. Polymorphism between the Parents

Of the 21 *Saltol*-linked markers tested, three markers, RM8094, RM493 and RM10793, were found polymorphic between the parents. Further, four markers were found polymorphic among the 42 tested on the flanking regions of the *Saltol*, resulting in a cumulative polymorphism of 11.1% on chromosome 1. Genome-wide polymorphism survey using 435 SSR markers (this included 42 markers tested on chromosome 1) identified a total of 62 polymorphic markers between PB1 and FL478, ranging from 4–7 markers spanned on each chromosome, resulting in an overall polymorphism of 14.7% between the parents (Table 1).

### 3.2. Development of Near Isogenic Lines by Marker-Assisted Selection

Five out of the 15 *F_1_* plants from the cross PB1/FL478 were found to be true *F_1_*s as they were heterozygous for the *Saltol*-linked marker RM493. One of the true *F_1_* plants was backcrossed with PB1 to produce 20 *BC_1_F_1_* plants. Foreground analysis using the three *Saltol-*linked markers identified six of the 20 plants to be heterozygous for all the three markers. These six *BC_1_F_1_* plants were phenotypically closer to PB1 than the other plants. Background analysis of these six plants using the 62 SSR markers indicated an average RPG recovery of 75.3% (range, 72.6 to 79.8%). One progeny with the highest RPG recovery (79.8%), Pusa 1822-6, was backcrossed with PB1 to generate 70 *BC_2_F_1_* seeds. In the *BC_2_F_1_* generation, 14 plants were heterozygous for all three *Saltol-*linked markers; these plants were subjected to phenotypic selection to identify six plants that were phenotypically closer to PB1 for agro-morphological and grain quality traits than the remaining plants. Background analysis of these six plants, using 21 unfixed markers in Pusa 1822-6, indicated RPG recovery from 86.3% to 89.5%, with an average of 87.8%. All the six plants were selfed to generate six *BC_2_F_2_* families. A total of 60 *BC_2_F_2_* plants, 10 plants each from a family, were subjected to foreground selection. Eleven *BC_2_F_2_* plants were found to be homozygous for the three *Saltol-*linked markers. Background analysis of these plants showed RPG recovery ranging from 89.9 to 92.7% with an average of 91.2%. These plants were further characterised for morphological and grain quality traits. Screening of eleven *BC_2_F_3_* families raised by selfing of the selected *BC_2_F_2_* plants for seedling stage salinity tolerance, identified one family, Pusa 1822-6-14-9, with a salt tolerance level comparable to that of FL478. All the plants of this family from the screening system were field transplanted to raise *BC_2_F_3_* population.

**Figure 2:**
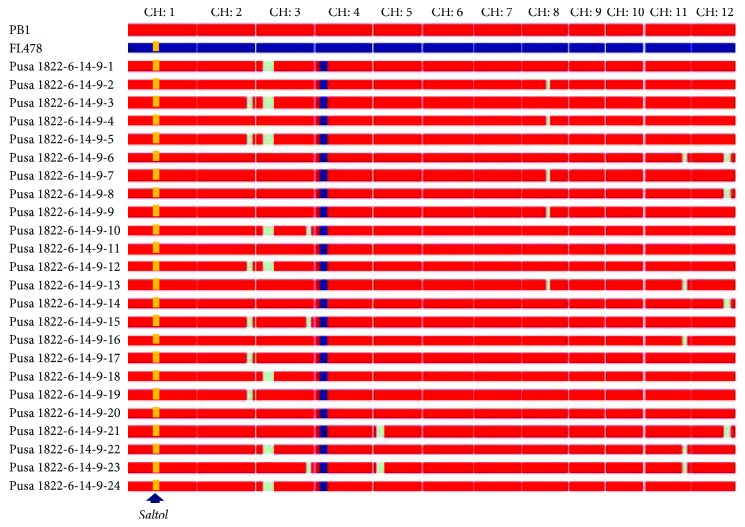
Graphical representation of the genotypes of 24 *Saltol*-introgressed NILs of PB1. The recurrent parent genome recovery ranged between 96.0 and 98.4%. All the NILs had maximum recovery on the carrier chromosome 1. CH: chromosome.

The *BC_2_F_3_* plants from Pusa 1822-6-14-9 were evaluated for both agro-morphological and grain quality traits, and 24 plants were selected for closer similarity with PB1. Background analysis of these plants, using six unfixed markers in the previous generation, indicated a cumulative RPG recovery of 96.0 to 98.4% (Table 2). All the 24 plants were advanced to *BC_2_F_4_* generation by selfing. No selection was done beyond *BC_2_F_4_* as all the *Saltol*-introgressed PB1 NILs had an average RPG recovery of more than 97%. Some residual donor segments were observed in chromosomes 2, 3, 5, 8, 11, and 12, whereas complete recovery was achieved in chromosomes 1, 6, 7, 9, and 10 (Figure 2). These lines are sequentially identified as NIL1 (Pusa 1822-6-14-9-1) to NIL24 (Pusa 1822-6-14-9-24).

### 3.3. Seedling Stage Salinity Tolerance

All the 24 NILs showed good seedling stage salinity tolerance (score of 1) comparable to that of FL478 under a salt stress of 11.6 dSm^−1^ (100 mM of NaCl) for sixteen days. In contrast, PB1 showed a highly sensitive reaction (score of 9) (Table 2; Supplementary Figure 1 [b]). The concentrations of major cations, Na^+^ and K^+^, that influence the salt response in rice seedlings are presented in Table 3. Broadly, there was significant variation among the NILs and their parents for cation contents and their ratios in both shoots and roots under both stressed as well as unstressed conditions. Under unstressed conditions, the root and shoot cation contents of the parental lines were comparable, but there were significant differences between some NILs, and some of them differed significantly from the parents as well. However, under stressed conditions, PB1 and FL478 had significantly distinct cation concentration both in shoots and roots; while the K^+^ levels in the shoots and roots of FL478 were much higher than those in PB1, the Na^+^ content in the shoots of FL478 was significantly lower than PB1, whereas Na^+^ content in the roots of PB1 was lower than FL478. All the NILs showed shoot and root K^+^ levels closer to those of FL478 than to PB1. The root Na^+^ concentration of NILs were closer to that of PB1, but shoot Na^+^ content was comparable or marginally lower than that of FL478. Further, there were several NILs that showed Na^+^/K^+^ ratio lower than that of FL478.

The correlations between cation content in shoots and roots (Table 4) under stressed and nonstressed conditions were insignificant. Under salt stress, salt tolerance score was found to have a significant positive association with shoot Na^+^ content, while shoot K^+^ level showed a negative association. Similar trend was observed for root ion concentrations under stress, except for root Na^+^ content, which exhibited nonsignificant correlation. The ionic proportions had shown very high negative association with salt tolerance score in both shoots and roots.

Correlations among the cation contents in shoots and roots under salt stress, indicated several significant associations such as a positive trend between shoot Na^+^ content and root Na^+^ content (0.38), as well as between shoot K^+^ and root K^+^ contents (0.57). There were no associations between shoot Na^+^ and root K^+^ levels and vice versa. Na^+^ content showed a major positive association with Na^+^/K^+^ ratio in both shoots and roots (0.66 and 0.47, resp.), while the K^+^ content showed significant negative association with the Na^+^/K^+^ ratio (−0.63 and −0.54, resp.). The cation ratios between shoots and roots also showed a positive trend (0.80). Further, cross associations were also noticed for shoot ion concentrations with root cation ratios (0.55 and −0.39, resp., for shoot Na^+^ and K^+^ contents), while root K^+^ showed a negative association with shoot Na^+^/K^+^ ratio (−0.52), but no such association was found with root Na^+^ content. Further, root Na^+^ and K^+^ contents showed a positive association (0.41).

### 3.4. Agronomic Performance

Mean performance of each of the 24 PB1 NILs for yield and yield-related traits is presented in Table 2. The NILs were essentially comparable to the recurrent parent, PB1, for agronomic traits, such as plant height, panicle length, weight of 1000 grains, and yield per plant. The days to 50% flowering ranged from 95.0 days (NIL19) to 106 days (NIL4, 6, 7, and 22): 23 NILs were at par with PB1 (103 days), while NIL19 was significantly flowering earlier than PB1.

### 3.5. Grain and Cooking Quality

**Figure 3:**
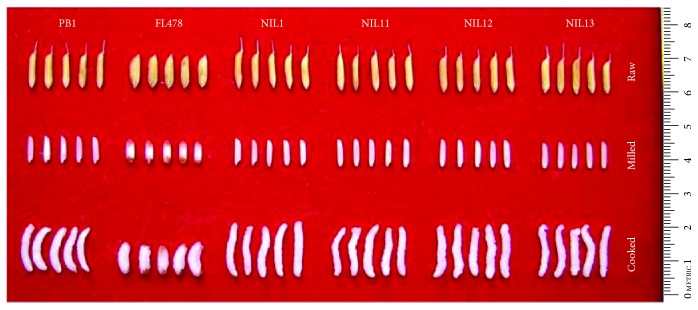
Grain and cooking quality of some of the NILs of Pusa Basmati 1 carrying *Saltol* locus.

The mean grain and cooking quality parameters of the NILs are presented in Table 5. Hulling and milling percentages for all the NILs were similar to those of the recurrent parent, PB1. Further, all the NILs possessed extra-long slender grain type (Figure 3) with strong aroma and with low gelatinization temperature as indicated by the alkali spreading value of 7.0, which is the same as that of PB1. Some of the NILs had significantly longer grain length before/after cooking than the RP while few had them significantly shorter, but these differences were rather small (0.15 mm or less).

## 4. Discussion

Growing demand for Basmati rice has resulted in its increased cultivation in the north-western areas of India [11, 12]. However, soil salinization in these regions poses a major threat to cropping as salinity stress leads to poor crop establishment and survival resulting in significant yield losses. Therefore, it is important to develop salt stress tolerant Basmati cultivars for cultivation in these areas [37]. In the present study, marker-assisted backcross breeding based on the established step-wise selection approach, namely, foreground, phenotypic, and background selections in the given order, was successful in improving the salt tolerance of PB1 Basmati rice variety. Stringent phenotypic selection carried out after the foreground selection is reported to accelerate RP genome recovery process [5, 11, 38, 39] and is expected to reduce the cost of background selection by reducing the number of test plants. It is noteworthy that very high (~96–98%) RPG recovery was achieved with only two backcrosses, and the recovery of the Basmati grain and cooking quality traits was almost complete. Further, there was complete recovery of the carrier chromosome (chromosome 1) together with *Saltol*, the target QTL (Figure 2; Supplementary Figure 1; Supplementary Table 1); this might have been facilitated by the relatively low level of polymorphism (11.1%) for this chromosome. This indicates the effectiveness of the selection procedure used in the study.

It is pertinent here to mention that PB1 was reported to possess a *Saltol* haplotype that was different from other Basmati cultivars. The PB1 haplotype shared a close homology with the *Saltol* locus of FL478, by differing only for three markers RM8094, RM493, and RM10793 [37]. Among these, RM8094 was the only recognised *Saltol*-linked marker that has been used for marker-assisted breeding, while RM493 and RM10793 were centromeric distal markers [20]. This implied that PB1 *Saltol* locus was very similar to FL478 locus, except for the region proximal to RM8094 marker locus. Therefore, the contrasting salt stress response between PB1 and FL478 can be arbitrarily assigned to a segment within 10.8 to 11.4 Mbp on chromosome 1. The NILs showed seedling stage salt tolerance levels comparable to that of FL478. Although, there was up to 4% residual donor genome present in some of the NILs, there was little effect of the donor genome on the agronomic performance, except for days to 50% flowering that was significantly lower than PB1 in one of the NILs, Pusa 1822-6-14-9-19.

Inspite of huge strides made in genomics-assisted breeding, development of salt-tolerant rice cultivars continues to be a major challenge due to the complex nature of *Saltol* region. Although, all the selected eleven *BC_2_F_2_* genotypes possessed the target marker alleles in homozygous condition, they exhibited differential tolerance response ranging from susceptibility to complete tolerant at 11.6 dSm^−1^ ECE level (Supplementary Table 3). The sensitive response of some *BC_2_F_2_* lines suggests the possibility that some genomic regions of PB1 may harbour genes/QTLs that have inhibitory effect on the *Saltol* QTL. Another possibility is cryptic intra-*Saltol* QTL recombination that could not be detected by the three markers used for the foreground selection. It is emphasised that *Saltol* region is fairly large (having a size of ~1.5Mbp) enough to accede intra-QTL recombination, as evident from its highly fragmented existence in the rice genome [40]. Further, introgression of additional hitherto unidentified QTLs from donor into the salt tolerant NILs cannot be ruled out.

The *Saltol* QTL region consists of several genes associated with salt response. These include transcription factors, signal transduction components, cell wall components, and membrane transporters [41, 42]. Specific genes, such as Na^+^ transporter gene *OsHKT1;5* [20, 43], osmoprotection-associated *SalT* [44], cation-proton exchanger (*OsCHX11*), cyclic nucleotide-gated ion channel (*OsCNGC1*) [45], high affinity potassium transporter (*HKT1*), and ATP-binding cassette transporter (*ABC1*) [42, 46], have been recognized in this region. However, since *Saltol* QTL is associated with Na^+^/K^+^ balance in the shoot tissues, the implicit mechanism of tolerance is attributed to Na^+^/K^+^ homeostasis driven by *OsHKT1;*5. The *OsHKT1;*5 gene, also known as *SKC1*, encodes for a xylem-expressed Na^+^ transporter and acts by preferentially unloading Na^+^ ions from xylem vessels while regulating K^+^ homeostasis [19]. Current observation of absence of any relation between the cation content between stressed and unstressed conditions indicated that ion homeostasis mechanisms might be active only under salt stress. Further, under stress, the shoot cation content outweighed root cation status in determining the salt tolerance, among which Na^+^ content was more deterministic of the level of tolerance than the K^+^ content. This strongly suggested Na^+^ transport as the major mechanism of salt tolerance in *Saltol*. The shift in Na^+^/K^+^ cation balance in shoot tissues of NILs towards the ratio in the donor parent FL478 tends to support this suggestion. Successful recovery of Basmati grain and cooking quality traits, together with pleasing aroma, were achieved in this study, as in several previous studies [1, 3, 4, 11, 39], in spite of the donor parent having poor grain and cooking quality traits. This was possible solely due to the marker-assisted selection strategy that combined a rigorous phenotypic selection in every generation.

## 5. Conclusions

In the present investigation, incorporation of seedling stage salinity tolerance in PB1 was achieved by introgression of the *Saltol* QTL using marker-assisted backcross breeding. The improved lines showed marked enhancement of salt tolerance in seedling stage. Since salt tolerance in Basmati cultivars is absent, the newly developed lines together with *Saltol*-introgressed NILs of Pusa Basmati 1121, another premium Basmati cultivar [11], will now offer choice of cultivars to be grown in salt-affected soils. Two of the improved NILs, Pusa 1822-6-14-9-11 and Pusa 1822-6-14-9-20 (Figure 3; Supplementary Figure 1 [c]), may be evaluated for their suitability for commercial cultivation and/or in breeding programmes for improving their reproductive stage salt tolerance, since the genetic controls of seedling and reproductive stage salt tolerance are different [47]. Additionally, a comprehensive evaluation of the NILs under salt-affected soil will reveal, other than agronomic performance, physiological improvements such as photosynthetic efficiency gained by incorporation of salt tolerance.

## Figures and Tables

**Table 1:** Genetic diversity between the recurrent parent PB1 and the *Saltol* donor FL478. The foreground survey was limited to *Saltol* region alone, whereas the background survey included all chromosomes, including the *Saltol* carrier chromosome 1.

Class of markers	Markers surveyed	Polymorphic markers	Polymorphism (%)
Foreground	21	3	14.29
Background^∗^	435	62	14.75
Chromosome 1^§^	63	7	11.11

∗ includes markers on chromosome 1, excluding *Saltol*-linked markers; ^§^Based on all markers used including *Saltol*-linked markers.

**Table 2:** Agronomic performance, salt tolerance, and recurrent parent genome recovery of *Saltol*-introgressed NILs of Pusa Basmati 1.

NILS	Agronomic traits								RPG recovery			
	DFF	PH	ETP	PL	SF	TW	YLD	STS	RP	HT	DP	RPG %
NIL1	101.0^a–e^	97.7^a^	20.7^ab^	27.8^a^	77.4^ab^	19.4^a^	43.6^a^	1.0	60	1	1	97.58
NIL2	98.5^c–e^	93.3^a^	17.7^ab^	28.4^a^	70.8^ab^	19.4^a^	43.4^a^	1.0	60	1	1	97.58
NIL3	97.0^de^	86.7^a^	14.6^ab^	25.3^a^	71.0^ab^	16.5^a^	38.7^a^	1.0	59	2	1	96.77
NIL4	106.0^a^	86.0^a^	14.1^ab^	27.2^a^	81.7^a^	19.9^a^	38.3^a^	1.0	60	1	1	97.58
NIL5	105.0^a–c^	88.9^a^	15.1^ab^	26.5^a^	74.1^ab^	17.8^a^	39.1^a^	1.0	59	2	1	96.77
NIL6	106.0^a^	95.6^a^	14.9^ab^	27.8^a^	78.7^ab^	19.8^a^	38.5^a^	1.0	59	2	1	96.77
NIL7	106.0^a^	91.5^a^	12.8^b^	26.9^a^	69.5^ab^	19.9^a^	39.0^a^	1.0	60	1	1	97.58
NIL8	98.5^c–e^	93.7^a^	18.9^ab^	29.0^a^	76.8^ab^	18.9^a^	40.2^a^	1.0	60	1	1	97.58
NIL9	98.5^c–e^	93.8^a^	14.3^ab^	26.9^a^	81.3^a^	18.0^a^	39.7^a^	1.0	60	1	1	97.58
NIL10	99.0^b–e^	90.3^a^	16.7^ab^	25.2^a^	79.2^a^	19.1^a^	38.9^a^	1.0	59	2	1	96.77
NIL11	103.0^a–d^	95.0^a^	17.6^ab^	28.7^a^	82.7^a^	18.9^a^	44.9^a^	1.0	61	0	1	98.39
NIL12	103.0^a–d^	88.0^a^	15.4^ab^	27.5^a^	78.0^ab^	19.4^a^	42.9^a^	1.0	59	2	1	96.77
NIL13	99.0^b–e^	91.2^a^	12.7^b^	27.5^a^	79.3^a^	18.1^a^	42.6^a^	1.0	59	2	1	96.77
NIL14	105.0^a–c^	97.6^a^	13.4^ab^	27.7^a^	76.5^ab^	19.9^a^	40.0^a^	1.0	60	1	1	97.58
NIL15	105.5^ab^	95.4^a^	15.2^ab^	27.2^a^	77.6^ab^	17.6^a^	41.3^a^	1.0	59	2	1	96.77
NIL16	100.5^a–e^	92.3^a^	17.6^ab^	25.1^a^	75.8^ab^	16.9^a^	39.0^a^	1.0	60	1	1	97.58
NIL17	102.0^a–d^	94.0^a^	17.9^ab^	25.8^a^	72.9^ab^	16.7^a^	41.6^a^	1.0	60	1	1	97.58
NIL18	99.0^b–e^	91.4^a^	18.0^ab^	27.0^a^	78.8^a^	17.5^a^	41.2^a^	1.0	60	1	1	97.58
NIL19	95.0^e^	93.8^a^	14.3^ab^	26.0^a^	78.3^ab^	19.6^a^	42.5^a^	1.0	60	1	1	97.58
NIL20	98.5^c–e^	96.2^a^	16.6^ab^	27.1^a^	80.2^a^	18.0^a^	40.3^a^	1.0	61	0	1	98.39
NIL21	102.0^a–d^	100.6^a^	22.5^a^	27.4^a^	73.3^ab^	19.4^a^	42.2^a^	1.0	59	2	1	96.77
NIL22	106.0^a^	99.7^a^	17.7^ab^	26.0^a^	58.4^b^	16.6^a^	37.2^a^	1.0	59	2	1	96.77
NIL23	100.0^a–e^	89.6^a^	17.5^ab^	27.0^a^	78.6^ab^	17.6^a^	40.4^a^	1.0	58	3	1	95.97
NIL24	104.0^a–c^	101.5^a^	14.7^ab^	28.1^a^	76.2^ab^	19.9^a^	41.6^a^	1.0	60	1	1	97.58
PB1	103.0^a–d^	98.9^a^	18.4^ab^	27.9^a^	76.8^ab^	19.5^a^	43.3^a^	9.0	100	0	0	—
FL478	83.5	97.0	13.0	25.0	85.5	26.2	45.5	1.0	0	0	100	—
CV (%)	1.56	5.52	13.80	4.00	6.50	6.85	5.36	—	—	—	—	
SE	1.59	5.17	2.26	1.08	4.95	1.27	2.19	—	—	—	—	

Means followed by same letters are statistically not different (*p* < 0.05), by Tukey's honest significance test. DFF: days to 50% flowering; PH: plant height in cm; ETP: effective tillers per plant; PL: panicle length in cm; TW: weight of 1000 grains in grams; YLD: yield in g per hill; STS: salt tolerance score (IRRI, 2013); RP: number of recurrent parent homozygotes; DP: number of donor parent homozygotes; HT: heterozygotes; RPG: recurrent parent genome recovery; CV: coefficient of variation; SE: standard error.

**Table 3:** Cation (Na^+^ and K^+^) content in the *Saltol*-introgressed PB1 lines and the donor and recipient parents under salt-stressed and salt-unstressed treatments.

NILs	Unstressed						Salt stressed					
	Shoot			Root			Shoot			Root		
	Na^+^	K^+^	Na^+^/K^+^	Na^+^	K^+^	Na^+^/K^+^	Na^+^	K^+^	Na^+^/K^+^	Na^+^	K^+^	Na^+^/K^+^
NIL1	2.8^a–f^^∗^	26.5^b–d^	0.11^a–f^	3.3^ij^	27.2^a–c^	0.12^j^	15.2^i–l^	23.6^a–d^	0.65^b^	20.7^d–f^	23.9^b–e^	0.86^e–i^
NIL2	2.7^a–f^	29.0^a–d^	0.09^b–f^	3.6^e–j^	17.7^k^	0.21^a–d^	13.8^j–l^	23.2^a–d^	0.60^b^	20.1^e–g^	20.8^g–i^	0.97^c–f^
NIL3	2.4^c–f^	28.9^a–d^	0.08^c–f^	5.1^a^	24.1^c–f^	0.21^a–c^	12.2^l^	22.0^a–e^	0.56^b^	19.5^e–h^	23.6^b–f^	0.82^e–j^
NIL4	3.4^a–e^	28.7^a–d^	0.12^a–f^	3.6^f–j^	18.0^k^	0.2^a–f^	24.5^b–d^	21.2^a–e^	1.16^b^	14.6^m–o^	15.1^lm^	0.98^b–e^
NIL5	2.1^ef^	32.4^a–d^	0.07^ef^	3.7^e–j^	21.9^f–i^	0.17^e–i^	23.0^c–f^	25.7^a–c^	0.89^b^	19.1^e–h^	26.3^b^	0.73^f–j^
NIL6	2.5^b–f^	28.7^a–d^	0.09^b–f^	3.4^g–j^	23.2^e–g^	0.15^ij^	22.4^d–f^	23.3^a–d^	0.96^b^	19.2^e–h^	23.0^c–g^	0.83^e–j^
NIL7	3.8^a–e^	26.8^b–d^	0.14^a–e^	4.7^a–d^	27.9^ab^	0.17^e–i^	27.0^bc^	25.0^a–d^	1.10^b^	20.9^de^	18.6^i–k^	1.13^b–d^
NIL8	2.7^a–f^	30.6^a–d^	0.09^b–f^	5.0^ab^	29.0^a^	0.17^d–i^	21.2^d–h^	22.5^a–d^	0.94^b^	11.0^q^	16.3^k–m^	0.68^g–j^
NIL9	3.5^a–e^	31.2^a–d^	0.11^a–f^	4.3^b–e^	18.2^jk^	0.23^a^	18.7^f–i^	21.6^a–e^	0.86^b^	24.0^c^	19.6^h–j^	1.22^b^
NIL10	4.3^a–c^	25.2^c–e^	0.18^a^	4.8^a–c^	27.2^a-c^	0.17^c–i^	12.4^kl^	12.2^e^	1.21^b^	18.2^g–j^	20.6^g–i^	0.88^d–i^
NIL11	4.5^ab^	30.3^a–d^	0.15^a–d^	4.1^c–g^	26.6^a–c^	0.16^g–j^	13.2^kl^	28.1^ab^	0.47^b^	14.0^no^	21.1^f–i^	0.66^h–j^
NIL12	2.4^c–f^	31.1^a–d^	0.08^d–f^	3.3^h–j^	19.0^i–k^	0.18^c–i^	13.9^j–l^	17.9^c–e^	0.78^b^	11.3^pq^	15.3^lm^	0.74^e–j^
NIL13	2.6^b–f^	17.7^e^	0.15^a–d^	4.7^a–d^	25.3^b–e^	0.19^b–h^	16.2^i–l^	21.3^a–e^	0.76^b^	11.3^pq^	16.7^k–m^	0.68^g–j^
NIL14	2.6^b–f^	26.7^b–d^	0.10^b–f^	4.0^d–h^	21.4^f–i^	0.19^b–h^	25.6^b–d^	24.7^a–d^	1.05^b^	13.4^op^	14.5^m^	0.92^d–g^
NIL15	2.8^a–f^	27.0^b–d^	0.11^a–f^	5.1^a^	21.9^f–i^	0.23^a^	27.1^bc^	24.6^a–d^	1.15^b^	26.7^b^	22.6^d–g^	1.18^bc^
NIL16	4.1^a–e^	28.5^a–d^	0.14^a–e^	3.0^j^	19.6^h–k^	0.16^g–j^	17.7^g–j^	15.5^de^	1.22^b^	13.8^no^	20.6^g–i^	0.67^h–j^
NIL17	3.1^a–f^	34.9^a^	0.09^b–f^	4.2^c–f^	22.9^e–g^	0.18^b–i^	21.5^d–g^	24.8^a–d^	0.87^b^	16.2^j–m^	22.9^d–g^	0.71^g–j^
NIL18	2.9^a–f^	32.9^a–c^	0.09^b–f^	4.2^c–f^	21.2^f–j^	0.2^a–e^	23.5^b–e^	22.9^a–d^	1.03^b^	18.7^f–i^	21.8^e–h^	0.86^e–i^
NIL19	4.4^ab^	32.0^a–d^	0.14^a–e^	4.2^c–f^	19.6^h–k^	0.22^ab^	24.9^b–d^	24.7^a–d^	1.01^b^	15.9^k–n^	24.9^b–d^	0.64^ij^
NIL20	1.3^f^	31.8^a–d^	0.04^f^	5.1^a^	26.8^a–c^	0.19^b–g^	16.9^h–k^	18.1^c–e^	0.94^b^	13.6^o^	17.5^j–l^	0.78^e–j^
NIL21	2.2^d–f^	33.8^ab^	0.07^ef^	3.9^e–i^	18.9^i–k^	0.20^a–e^	22.6^c–f^	22.7^a–d^	0.99^b^	19.1^e–h^	21.2^f–i^	0.90^d–h^
NIL22	4.1^a–d^	24.9^de^	0.17^ab^	4.2^c–f^	23.4^d–f^	0.18^c–i^	22.1^d–g^	18.6^b–e^	1.20^b^	17.0^i–l^	19.8^h–j^	0.86^e–i^
NIL23	4.6^a^	29.0^a–d^	0.16^a–c^	4.1^c–g^	26.5^a–d^	0.15^h–j^	22.9^c–f^	26.9^a–c^	0.85^b^	15.4^l–o^	25.6^bc^	0.60^j^
NIL24	4.3^a–c^	32.8^a–c^	0.13^a–e^	3.5^g–j^	21.3^f–j^	0.16^f–i^	19.4^e–i^	23.4^a–d^	0.83^b^	17.8^h–k^	20.4^g–i^	0.87^e–i^
PB1	3.2^a–f^	25.5^cd^	0.13^a–e^	3.8^e–i^	20.1^g–k^	0.19^b–h^	34.4^a^	12.2^e^	2.83^a^	22.3^cd^	8.2^n^	2.72^a^
FL478	3.6^a–e^	28.6^a–d^	0.13^a–e^	5.2^a^	22.3^e–h^	0.23^a^	27.9^b^	29.4^a^	0.95^b^	29.3^a^	30.4^a^	0.96^c–f^
CV (%)	15.25	6.48	17.33	4.12	3.33	4.82	5.30	10.84	19.34	2.88	3.17	6.57
SE	0.49	1.88	0.02	0.17	0.76	0.01	1.10	2.40	0.19	0.51	0.65	0.06

^∗^Means followed by the same letter are statistically not different at *p* < 0.05, by Tukey's honest significance test. CV: coefficient of variation; SE: standard error.

**Table 4:** Interrelationships of cation content and their proportions in root and shoots under unstressed (lower diagonal) and salt-stressed (upper diagonal) conditions. Cross correlations between unstressed and stressed conditions are given as diagonal elements.

Parameters^†^	St: Na^+^	St: K^+^	St: Na^+^/K^+^	Rt: Na^+^	Rt: K^+^	Rt: Na^+^/K^+^	STS
St: Na^+^	0.072	0.101	0.658^∗^	0.380^∗^	−0.130	0.545^∗^	0.494^∗^
St: K^+^	−0.063	0.171	−0.625^∗^	0.186	0.567^∗^	−0.389^∗^	−0.437^∗^
St: Na^+^/K^+^	0.864^∗^	−0.534^∗^	0.107	0.197	−0.519^∗^	0.801^∗^	0.833^∗^
Rt: Na^+^	−0.105	−0.161	0.013	0.227	0.405^∗^	0.465^∗^	0.201
Rt: K^+^	0.052	−0.250	0.176	0.480^∗^	0.115	−0.537^∗^	−0.551^∗^
Rt: Na^+^/K^+^	−0.135	0.086	−0.148	0.521^∗^	−0.490^∗^	0.244	0.908^∗^

^†^St: Shoot; Rt: Root; ^∗^Correlation coefficients are significant at *p* < 0.01 level; STS: salt tolerance score.

**Table 5:** Grain and cooking quality of *Saltol*-introgressed NILs of Pusa Basmati 1 (Pusa 1822).

NILS	HUL	MIL	KLBC	KLAC	ER	ASV	AROM
NIL1	75.5^a^	66.9^a^	7.29^a^	13.32^a^	1.822^de^	7.0	2.0
NIL2	73.7^a^	65.3^a^	7.21^d–g^	13.21^b–d^	1.832^c–e^	7.0	2.0
NIL3	74.7^a^	68.7^a^	7.27^a–c^	13.24^bc^	1.819^ef^	7.0	2.0
NIL4	72.8^a^	64.7^a^	7.20^e–h^	13.21^b–d^	1.837^a–c^	7.0	2.0
NIL5	72.7^a^	64.6^a^	7.22^d–f^	13.21^b–d^	1.825^de^	7.0	2.0
NIL6	74.8^a^	66.0^a^	7.19^f–i^	13.12^fg^	1.822^de^	7.0	2.0
NIL7	69.7^a^	62.9^a^	7.23^c–f^	13.20^cd^	1.827^c–e^	7.0	2.0
NIL8	74.0^a^	66.7^a^	7.29^ab^	13.26^b^	1.819^ef^	7.0	2.0
NIL9	74.2^a^	66.5^a^	7.25^b–d^	13.22^bc^	1.822^de^	7.0	2.0
NIL10	73.5^a^	66.5^a^	7.19^f–i^	13.20^cd^	1.836^a–c^	7.0	2.0
NIL11	75.6^a^	67.5^a^	7.29^ab^	13.31^a^	1.826^c–e^	7.0	2.0
NIL12	74.7^a^	66.2^a^	7.28^ab^	13.07^g^	1.795^g^	7.0	2.0
NIL13	73.4^a^	65.7^a^	7.21^d–g^	13.21^b–d^	1.830^c–e^	7.0	2.0
NIL14	76.4^a^	67.9^a^	7.20^e–h^	13.19^cd^	1.832^c–e^	7.0	2.0
NIL15	73.7^a^	65.6^a^	7.16^hi^	13.15^ef^	1.837^b–d^	7.0	2.0
NIL16	73.5^a^	67.6^a^	7.27^a–c^	13.25^bc^	1.823^ef^	7.0	2.0
NIL17	73.5^a^	67.7^a^	7.24^c–e^	13.00^h^	1.796^g^	7.0	2.0
NIL18	74.2^a^	66.9^a^	7.17^g–i^	13.23^bc^	1.845^a^	7.0	2.0
NIL19	76.5^a^	68.7^a^	7.22^d–f^	13.21^b–d^	1.835^c–e^	7.0	2.0
NIL20	73.6^a^	66.5^a^	7.21^d–g^	13.18^de^	1.828^c–e^	7.0	2.0
NIL21	73.4^a^	65.6^a^	7.20^e–h^	13.21^b–d^	1.837^a–c^	7.0	2.0
NIL22	71.9^a^	64.9^a^	7.20^e–h^	13.20^cd^	1.835^c–e^	7.0	2.0
NIL23	74.3^a^	65.1^a^	7.15^i^	13.20^cd^	1.846^a^	7.0	2.0
NIL24	73.4^a^	71.6^a^	7.15^i^	13.20^cd^	1.844^ab^	7.0	2.0
PB1	73.9^a^	66.0^a^	7.30^a^	13.21^b–d^	1.810^fg^	7.0	2.0
FL478	79.1	64.4	6.28	9.13	1.454	5.0	0.0
CV (%)	2.14	3.60	0.14	0.09	0.18	—	—
SE	1.58	2.39	0.01	0.01	0.00	—	—

Means followed by same letters are statistically not different at *p* < 0.05, by Tukey's honest significance test. HUL: hulling recovery in percentage; MIL: milling recovery in percentage; KLBC: kernel length before cooking in mm; KLAC: kernel length after cooking in mm; ASV: alkali spreading value; AROM: aroma score from panel test; CV: coefficient of variation; SE: standard error.

## Supplementary Materials

Supplementary Materials Supplementary Table 1: chromosome wise list of polymorphic STMS markers between PB1 and FL478. Supplementary Table 2: information on *Saltol*-linked SSR markers on chromosome1 polymorphic between PB1 and FL478. Supplementary Table 3: agronomic performance, salt tolerance, and recurrent parent genome recovery of *Saltol-*positive homozygous *BC_2_F_2_* plants. Supplementary Figure 1: phenotypic evaluation for salt tolerance between parents and introgressed lines with their field view. Supplementary Figure 2: screening of foreground markers in the parental lines. Three markers RM8094, RM493, and RM10793 showed polymorphism between PB1 and FL478.
