# Supplementary material for: Marker-Assisted Introgression of Saltol QTL Enhances Seedling Stage Salt Tolerance in the Rice Variety “Pusa Basmati 1”
Source: Int J Genomics. 2018 Feb 11;2018:8319879. doi: 10.1155/2018/8319879 (PMC5896403; doi:10.1155/2018/8319879)
Supplement: Supplementary Materials — Supplementary Table 1: chromosome wise list of polymorphic STMS markers between PB1 and FL478. Supplementary Table 2: information on Saltol-linked SSR markers on chromosome1 polymorphic between PB1 and FL478. Supplementary Table 3: agronomic performance, salt tolerance, and recurrent parent genome recovery of Saltol-positive homozygous BC2F2 plants. Supplementary Figure 1: phenotypic evaluation for salt tolerance between parents and introgressed lines with their field view. Supplementary Figure 2: screening of foreground markers in the parental lines. Three markers RM8094, RM493, and RM10793 showed polymorphism between PB1 and FL478. [file 8319879.f1.pdf]

Supplementary materials

Marker-assisted introgression of *Saltol* QTL enhances seedling stage salt tolerance in the rice variety 'Pusa Basmati 1'

Vivek Kumar Singh, Brahma Deo Singh, Amit Kumar, Sadhna Maurya, Subbiayan Gopala Krishnan, Kunnummal Kurungara Vinod, Madan Pal Singh, Ranjith Kumar Ellur, Prolay Kumar Bhowmick and Ashok Kumar Singh

Correspondence should be addressed to Ashok Kumar Singh; aks_gene@yahoo.com

SUPPLEMENTARY TABLE 1. Chromosome wise list of polymorphic STMS markers between PB1 and FL478

Marker No.	Polymorphic Markers	Physical position (Mb)	Number of Marker used	Marker coverage Mb/marker	% polymorphism
	**Chromosome 1**				
**1**	HVSSR01-32	9.61	42	10.83	9.50
**2**	RM14	25.65			
**3**	RM84	31.50			
**4**	HVSSR01-79	39.34			
	**Chromosome 2**				
**5**	HVSSR02-01	0.12	42	7.18	11.90
**6**	RM475	12.08			
**7**	RM262	20.79			
**8**	HVSSR02-68	27.95			
**9**	RM240	32.39			
	**Chromosome 3**				
**10**	RM3716	6.83	30	6.06	20.00
**11**	HVSSR03-37	15.03			
**12**	RM564	19.22			
**13**	RM16	23.89			
**14**	RM168	28.86			
**15**	HVSSR03-86	32.85			
	**Chromosome 4**				
**16**	RM3892	1.12	31	5.91	19.35
**17**	RM7200	4.03			
**18**	HVSSR04-15	11.39			
**19**	HVSSR04-28	21.50			
**20**	RM3387	24.68			
**21**	RM131	35.01			
	**Chromosome 5**				
**22**	RM437	3.87	21	6.00	23.80
**23**	HVSSR5-25	9.12			
**24**	RM6645	15.02			
**25**	RM3476	23.84			
**26**	RM31	28.75			

Marker No.	Polymorphic Markers	Physical position (Mb)	Number of Marker used	Marker coverage Mb/marker	% polymorphism
	**Chromosome 6**				
**27**	RM204	3.17	31	6.26	16.12
**28**	RM19973	13.42			
**29**	HVSSR06-40	17.35			
**30**	RM3	20.38			
**31**	HVSSR06-56	26.55			
	**Chromosome 7**				
**32**	RM21384	10.72	38	7.43	10.52
**33**	RM7110	16.85			
**34**	RM1279	22.28			
**35**	RM21976	25.59			
	**Chromosome 8**				
**36**	RM337	0.15	21	5.68	23.80
**37**	RM3481	9.14			
**38**	HVSSR08-29	15.83			
**39**	RM3153	19.08			
**40**	RM80	24.57			
	**Chromosome 9**				
**41**	RM5688	1.66	28	3.83	21.42
**42**	HVSSR09-07	4.92			
**43**	RM23914	7.24			
**44**	RM3912	10.83			
**45**	HVSSR09-36	17.47			
**46**	RM205	22.72			
	**Chromosome 10**				
**47**	RM271	3.48	34	4.64	14.70
**48**	RM25161	7.95			
**49**	RM311	9.74			
**50**	RM184	16.35			
**51**	RM590	23.04			
	**Chromosome 11**				
**52**	RM1124	3.84	45	4.83	13.33
**53**	RM26499	11.00			
**54**	RM7226	16.34			
**55**	RM229	20.70			
**56**	RM1341	22.01			
**57**	RM330	25.15			
	**Chromosome 12**				
**58**	RM7619	4.83	32	5.50	15.62
**59**	RM2935	7.43			
**60**	RM3137	8.16			
**61**	RM1261	17.68			
**62**	RM463	22.09			

SUPPLEMENTARY TABLE 2. Information on *Saltol* linked SSR markers on chromosome1 polymorphic between PB1 and FL478

Marker	FL478 allele	PB1 allele	Primer sequences	Motif	T_a	AL_e	Position (Mb)
RM8094	220 bp	175 bp	F: AAGTTTGTACACATCGTATACA	(AT)31	55	209	11.2
			R: CGCGACCAGTACTACTACTA				
RM493	250 bp	230 bp	F: TAGCTCCAACAGGATCGACC	(CTT)9	55	211	12.2
			R: GTACGTAAACGCGGAAGGTG				
RM10793	150 bp	130 bp	F: ACTTGCCAACTCCTTCAATTCG	(ATAG)7	55	124	12.5
			R: TCGTCGAGTAGCTTCCCTCTCTACC				

T_a, annealing temperature in °C; AL_e, expected amplicon length in bp (Thomson et al. 2010)

SUPPLEMENTARY TABLE 3. Agronomic performance, salt tolerance and recurrent parent genome recovery of *Saltol* positive homozygous BC_2F_2 plants

No.	Plant ID	Phenotypic selection					Foreground selection			Background selection				STS
		**PH**	**NT**	**PL**	**FG/P**	**TW**	**RM8094**	**RM493**	**RM10793**	**RP**	**H**	**DP**	**RPG%**	
**1**	PUSA 1822-6-1-4	101.3	21.0	21.9	140.0	23.4	++	++	++	53	8	1	91.94	7.0
**2**	PUSA 1822-6-1-10	102.4	23.0	23.2	139.0	19.8	++	++	++	54	7	1	92.74	7.0
**3**	PUSA 1822-6-4-2	100.9	19.0	19.0	133.0	24.2	++	++	++	54	5	3	91.13	7.0
**4**	PUSA 1822-6-4-8	102.1	21.0	21.5	132.0	21.4	++	++	++	54	5	3	91.13	7.0
**5**	PUSA 1822-6-6-6	101.2	22.0	19.8	135.0	19.9	++	++	++	53	6	3	90.32	5.0
**6**	PUSA 1822-6-6-15	102.7	19.0	22.5	134.0	21.5	++	++	++	54	5	3	91.13	7.0
**7**	PUSA 1822-6-9-13	103.6	21.0	24.2	130.0	23.9	++	++	++	53	6	3	90.32	7.0
**8**	PUSA 1822-6-11-4	103.3	18.0	18.9	137.0	21.7	++	++	++	54	5	3	91.13	5.0
**9**	PUSA 1822-6-11-5	103.7	24.0	25.7	140.0	22.3	++	++	++	52	7	3	89.52	7.0
**10**	PUSA 1822-6-11-11	103.9	19.0	22.3	138.0	24.2	++	++	++	53	6	3	90.32	7.0
**11**	PUSA 1822-6-14-9	102.4	23.0	21.1	137.0	23.3	++	++	++	54	7	1	92.74	1.0
**RP**	PB1	101.4	25.0	23.5	145.0	23.7	--	--	--	62	0	0	100.0	9.0
**DP**	FL478	97.3	12.0	24.0	122.0	25.0	++	++	++	0	0	62	0.0	1.0
	SD±	1.7	3.3	2.1	5.7	1.7								
	SE±	0.5	0.9	0.6	1.6	0.5								

PH, plant height in cm; NT, number of tillers/ plant; PL, panicle length in cm; FG/P, filled grain/ panicle; TW, thousand grain weight in g; RP, Recurrent Parent; DP, Donor Parent; H, Heterozygous; RPG, Recurrent Parent Genome recovery; STS, salt tolerance score

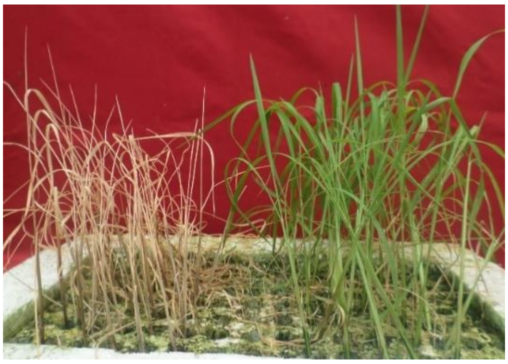

PB1 FL478

(a) Phenotypic evaluation of recurrent parent (RP) and donor parent (DP) for salinity tolerance

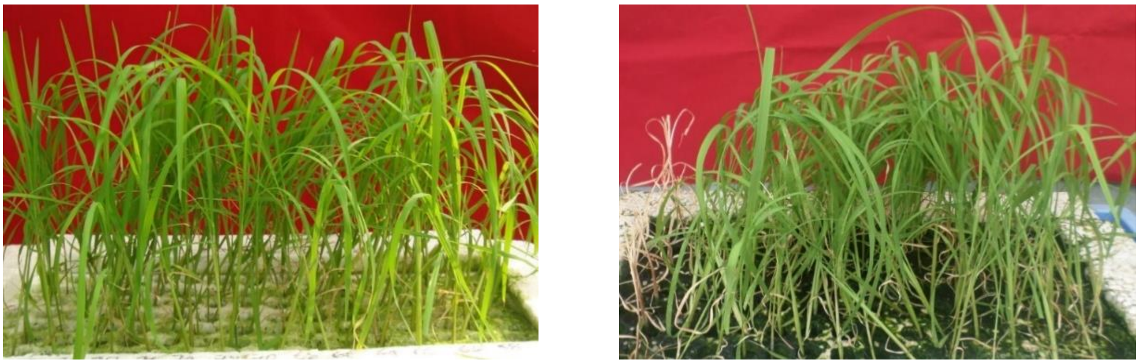

RP DP 1 → 12 RP DP 1 → 12

Unstressed Stressed

(b) Screening of *Saltol* positive homozygous BC_2F_3 plants for salinity tolerance

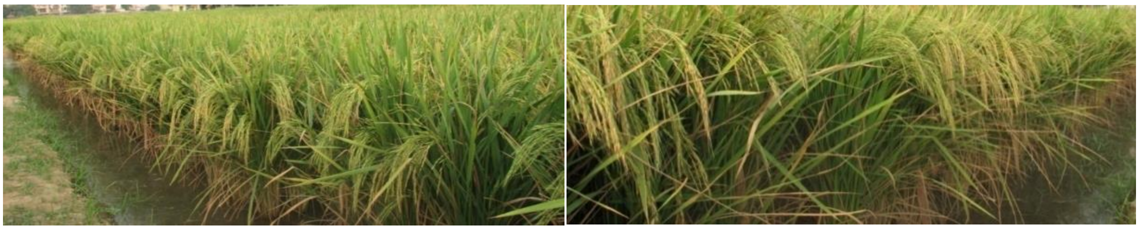

(c) Field view of agronomic evaluation of PB1 NILs, derived from Pusa 1822-6-14-9 with high level of salt tolerance

SUPPLEMENTARY FIGURE 1. Phenotypic evaluation for salt tolerance between parents and introgressed lines with their field view.

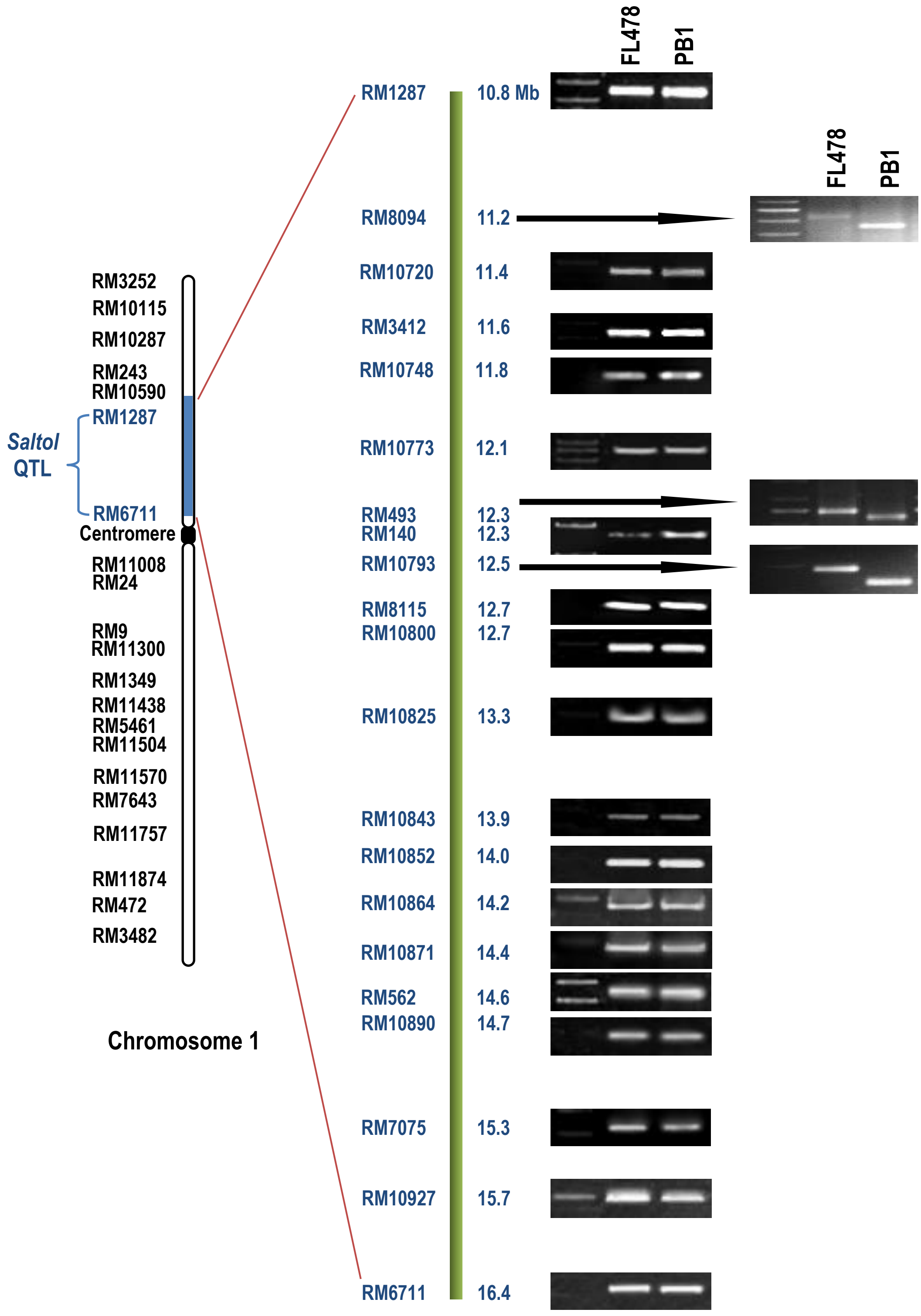

SUPPLEMENTARY FIGURE 2. Screening of foreground markers in the parental lines. Three markers RM8094, RM493 and RM10793 showed polymorphism between PB1 and FL478.
